# Correspondence: Analytical flaws in a continental-scale forest soil microbial diversity study

**DOI:** 10.1038/ncomms15572

**Published:** 2017-06-06

**Authors:** Leho Tedersoo

**Affiliations:** 1 Natural History Museum, University of Tartu, 14a Ravila, 50411 Tartu, Estonia

Nature Communications 8: Article number: 15572; DOI: 10.1038/ncomms15572 (2017); Published: 06 06 2017

Zhou *et al*.^1^ recently addressed continental-scale biogeographic patterns of soil microorganisms. The authors suggested that the mean annual temperature rather than other environmental predictors drive the latitudinal gradient of diversity in soil bacteria and fungi sampled from six permanent study areas across North America. Here I argue that their approach is flawed because of pseudoreplicated design, incorrect choice of statistical tools and overoptimistic interpretation.

From each of the six sites, the authors collected 21 composite soil cores (located 1–400 m apart) that were treated as independent sampling units to infer continental-scale patterns. The authors then used simple regression analyses to address the differences among samples instead of nesting samples within sites or using sites as categorical predictors, thus overestimating the residual degrees of freedom by 31-fold. Further, the authors calculated Chao1 minimum richness estimates for each site as proxies of diversity to test the response of richness to environmental variables. However, the minimum richness estimates are based on relative abundance of the rare taxa^2^. Most of this ‘rare biosphere' in metabarcoding studies (such as Zhou *et al*.^1^) may represent unfiltered analytical artefacts^3^,^4^ and magnify the differences in richness among taxon-poor and taxon-rich sites^4^. After integrating richness into Chao1 estimates, the authors conducted simple and multiple regression analyses for many explanatory variables, determining that inversely transformed temperature represents the best linear predictor for microbial richness. While this holds true for the current very limited sampling on a continental scale, inclusion of soils from broader range of pH and precipitation are likely to alter this pattern.

Previous research has indicated that on global and continental scales, soil pH and precipitation constitute the strongest predictors of richness and composition of terrestrial microorganisms^5^,^6^,^7^,^8^,^9^,^10^. Several of these studies have also identified temperature as one of the key predictors. Furthermore, two global studies previously reported that temperature is the strongest predictor of bacterial^11^ and fungal^12^ richness. Zhou *et al*.^1^ neglected to cite these studies, and thus over-emphasized the novelty of connecting temperature to the latitudinal gradient of diversity in microbes.

It has been well established that true replication is a cornerstone of experimental design in ecology^13^ and microbiology^4^,^14^ to secure correct data analysis and interpretation. Proper replication as well as spatially independent and representative samples is required to infer both small-scale and large-scale biogeographic patterns and processes. Samples that serve as good replicates on a fine scale may be, however, unsuited for this purpose over large geographic scales without introducing nested design or accounting for spatial autocorrelation. Therefore, I urge researchers to carefully consider replication on a relevant geographic scale in the discipline of biogeography.

## Additional information

**How to cite this article:** Tedersoo, L. Analytical flaws in a continental-scale forest soil microbial diversity study. *Nat. Commun.* **8,** 15572 doi: 10.1038/ncomms15572 (2017).

**Publisher's note**: Springer Nature remains neutral with regard to jurisdictional claims in published maps and institutional affiliations.
